# Polylactide Conjugates of Camptothecin with Different Drug Release Abilities

**DOI:** 10.3390/molecules191219460

**Published:** 2014-11-25

**Authors:** Ewa Oledzka, Paweł Horeglad, Zuzanna Gruszczyńska, Andrzej Plichta, Grzegorz Nałęcz-Jawecki, Marcin Sobczak

**Affiliations:** 1 Department of Inorganic and Analytical Chemistry, Faculty of Pharmacy, Medical University of Warsaw, Banacha 1, Warsaw 02-097, Poland; E-Mails: eoledzka@wp.pl (E.O.); zuzannagruszczynska@yahoo.com (Z.G.); 2 Centre of New Technologies, University of Warsaw, Banacha 2c, Warsaw 02-097, Poland; E-Mail: phoreglad@uw.edu.pl; 3 Chair of Polymer Chemistry and Technology, Faculty of Chemistry, Warsaw University of Technology, Noakowskiego 3, Warsaw 00-664, Poland; E-Mail: aplichta@ch.pw.edu.pl; 4 Department of Environmental Health Science, Faculty of Pharmacy, Medical University of Warsaw, Banacha 1, Warsaw 02-097, Poland; E-Mail: gnalecz@wum.edu.pl; 5 Chair of Chemistry, Department of Organic Chemistry, Faculty of Materials Science and Design, Kazimierz Pulaski University of Technology and Humanities in Radom, Chrobrego 27, Radom 26-600, Poland

**Keywords:** macromolecular conjugates, biodegradable polymers, camptothecin, drug delivery systems, controlled release

## Abstract

Camptothecin-polylactide conjugates (CMPT-PLA) were synthesized by covalent incorporation of CMPT into PLA of different microstructure, *i.e.*, atactic PLA and atactic-*block*-isotactically enriched PLA (*P*_m_ = 0.79) via urethane bonds. The kinetic release of CPMT from CMPT-PLA conjugates, tested *in vitro* under different conditions, is possible in both cases and notably, strongly dependent on PLA microstructure. It shows that release properties of drug-PLA conjugates can be tailored by controlled design of the PLA microstructure, and allow in the case of CMPT-PLA conjugates for the development of highly controlled biodegradable CMPT systems—important delivery systems for anti-cancer agents.

## 1. Introduction

Camptothecin ((4*S*)-4-ethyl-4-hydroxy-1*H*-pyrano[3',4':6,7]indolizino[1,2-b]quinoline-3,14(4*H*,12*H*)-dione, CMPT) belongs to the monoterpene indole alkaloid family, isolated from the tree *Camptotheca acuminata* [1]. CMPT and its derivatives like irinotecan and topotecan, commonly used in antitumor therapy, exhibit a broad range of antitumor and antileukaemia activity leading to the inhibition of topoisomerase I, subsequent damage of DNA and thus cell death, making them very effective in the treatment of multi-organ tumors. However, the clinical use of CMPT is limited by its low solubility in water, high toxicity and inactivation through lactone ring hydrolysis at a physiological pH [2]. These disadvantages could be overcome by attaching CMPT to a polymeric matrix. The polymeric conjugates of CMPT could act as a transport form for this drug and enhance its biodistribution [3]. In the past few years, CMPT has been covalently coupled to water-soluble polymers, *i.e.*, poly(ethylene glycol), poly(l-glutamic acid), β-cyclodextrin-based polymers, poly[*N*-(2-hydroxypropyl)-methacrylamide], poly[(*N*-carboxybutyl)-l-aspartamide] and poly(amido-amine). The development of such conjugates could stabilize the active form of the lactone ring of CMPT as well as improve its water solubility [2,4,5,6,7,8,9,10,11,12]. Although covalent conjugates of polylactide (PLA), a biodegradable and biocompatible polymer with a wide range of applications are known, PLA has not been applied for the synthesis of drug-PLA conjugates, which is due to its physicochemical properties; e.g., poly(l-lactide) (PLLA) is highly crystalline and degrades slowly [13,14]. In order to modify the drug release properties of PLA-based conjugates, PLA copolymers with poly(ε-caprolactone) (PCL), polyglycolide (PGL) and poly(trimethylenecarbonate) (PTMC) have been recently synthesized by us and used as polymeric matrices for the preparation of CMPT conjugates [3].

Although the properties of PLA depend strongly on its tacticity, the modification of microstructure of PLA in order to tailor its drug release properties has not been investigated so far, which results from the difficulty in modifying the PLA microstructure in a controlled way. To date a number of catalysts have allowed for the synthesis of atactic, isotactic (including isotactic stereoblock PLA), heterotactic or syndiotactic PLA in the polymerization of *rac*-lactide (*rac*-LA) or *rac*-lactide (*meso*-LA) [15,16] and recently isotactic-heterotactic stereomultiblock PLA [17], but fully controlled synthesis of PLA of a desired microstructure requires a catalyst which allows for easy switch of stereoselectivity, as we recently demonstrated for the catalytic system based on dialkylgallium alkoxides [18,19]. The latter allowed for the first time for the facile synthesis of PLA comprised of blocks of different tacticity, and showed that such a modification influences considerably the mechanical properties of PLA [19], and therefore could also influence the drug release profile.

In this regard, we decided to investigate whether the drug release properties of PLA-drug conjugates depend on the microstructure of PLA. Using CMPT-(atactic PLA)_100_ and CMPT-(atactic PLA)_50_-*b*-(isotactic PLA-*P*_m_=0.79)_50_ conjugates, hereby we demonstrate, for the first time, that CMPT-PLA conjugates can be used for the controlled delivery of CMPT, while the CMPT release is strongly dependent on the PLA microstructure.

## 2. Results and Discussion

In our previous paper [3] biodegradable conjugates of CMPT obtained from various copolymers were described. The copolymers were synthesized from ε-caprolactone (ε-CL), glycolide (GL), *rac*-lactide (*rac*-LA) or trimethylene carbonate (TMC) and poly(ethylene glycol) (PEG). It was found that the rates of CMPT release depended on the nature of the copolymers used in the matrices synthesis. Now, we wanted to check whether the microstructure of polylactides (PLAs) substantially influences the release kinetics of CMPT from the matrices.

**Scheme 1:**
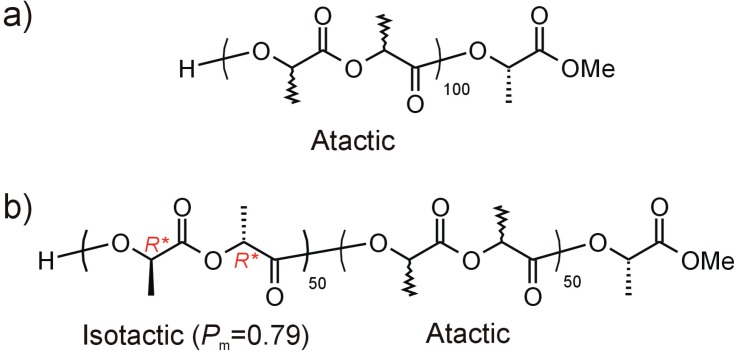
(atactic-PLA)_100_ (**PLA-1**) (**a**) and (atactic PLA)_50_*-b-*(isotactic PLA-*P*_m_=0.79)_50_ (**b**).

In the first step (Scheme 1a), (atactic-PLA)_100_ (**PLA-1**) was synthesized, according to the literature, by the ring opening polymerization (ROP) of *rac*-LA in the presence of dimethylgallium alkoxide (*S,S*)-[Me_2_Ga(μ-OCH(Me)CO_2_Me)]_2_ [20]. For the synthesis of (atactic PLA)_50_*-b-*(isotactic PLA-*P*_m_=0.79)_50_ (**PLA-2**) (Scheme 1b) the (*S,S*)-[Me_2_Ga(μ-OCH(Me)CO_2_Me)]_2_/DBU catalytic system was applied [19]. *M*_n_ and tacticity of **PLA-1** and **PLA-2** were confirmed by ^1^H-, ^13^C-NMR and homonuclear decoupled ^1^H-NMR (Figures S1–S6, Supplementary Materials), which was in agreement with literature data and confirmed the controlled nature of polymerization leading to **PLA-1** (Scheme 1a) and **PLA-2** (Scheme 1b). Of note, in light of the synthesis of PLA-CMPT conjugates, is the fact that the OH group used for the attachment of CMPT is connected in the case of **PLA-1** to atactic PLA, while for **PLA-2** it is to the isotactically enriched PLA block (Scheme 1).

In consideration of the potential biomedical applications of the obtained polymers, the problem of the toxicity resulting e.g., from the presence of catalyst residues is very important [21]. Thereby, the analysis of residual gallium content was determined using ASA and this revealed a residual gallium content of about 1 ppm. This is very satisfying result, as accordance with the guidelines of the European Pharmacopeia, whereby the metal content of polymers in contact with blood or blood components must not exceed 20 ppm.

The cytotoxicity of the synthesized PLAs were evaluated with bacterial luminescence (*V. fischeri*) test and two protozoan assays (*S. ambiguum* and *T. termophila*). It was found that both PLAs were not toxic to the tested organisms in direct contact and extraction tests as the percent of endpoints was lower than 20% (Table 1).

**Table 1:** Toxicity of tested polymers.

Sample	Spirotox		Protoxkit F		Microtox	
	DCT	EX	DCT	EX	DCT	EX
**PLA-1**	0	0	1	4	−4	0
**PLA-2**	0	0	6	10	−5	1

DCT—direct contact test; EX—tests with extracts.

**Scheme 2:**
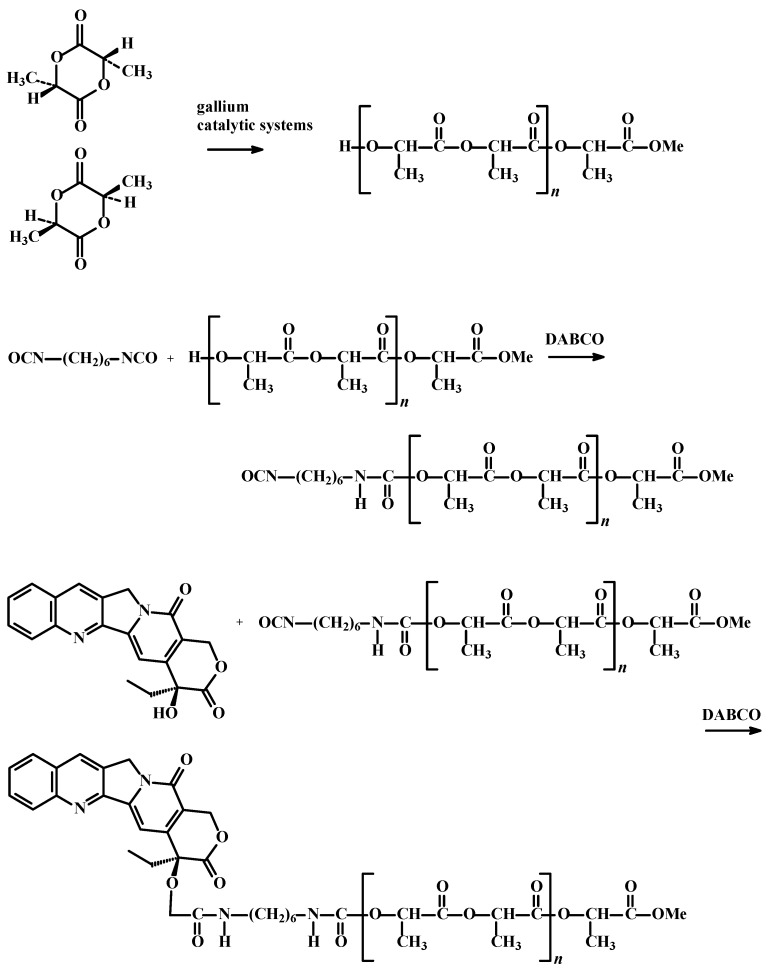
The synthesis of polylactides and macromolecular conjugates of CMPT.

In the next step, the polymeric conjugates of CMPT were obtained. The PLAs and CMPT were coupled via 1,6-diisocyanatohexane (HMDI) linker (Scheme 2).

**Figure 1:**
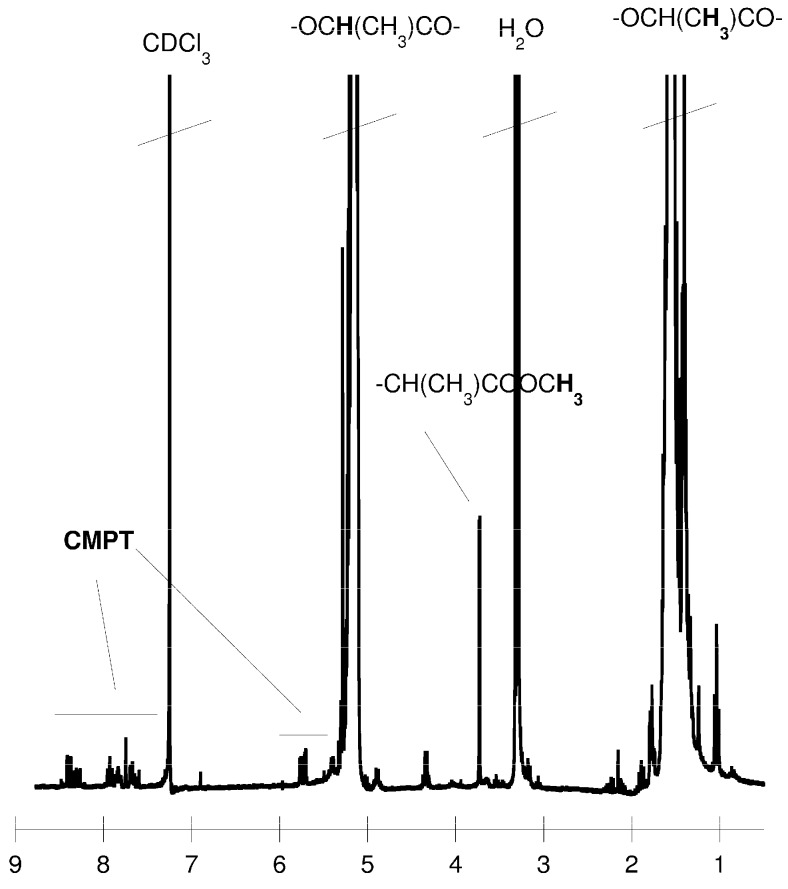
^1^H-NMR spectrum of the CMPT-PLA conjugate.

The structure of the CMPT-PLA conjugates was characterized by ^1^H-NMR spectroscopy, as presented in Figure 1. The characteristic signals of CMPT (without the δ = 6.30 ppm hydroxyl group, which has reacted with the isocyanate group) indicates the successful formation of the CMPT-PLA conjugates. The chemical shifts at 5.15 and 1.57 ppm are assigned to the protons in PLA, while the signals at 8.45, 8.17, 8.13, 7.87, 7.72, 7.36, 5.43, 5.30, 1.88 and 0.86 ppm are associated with CMPT. The CMPT content in conjugates was about 2 mol %, as estimated by the comparison of the intensity of signals corresponding to aromatic protons (8.45 ppm) (for the CMPT) and methylene protons (5.15 ppm) (for the PLA).

**Figure 2:**
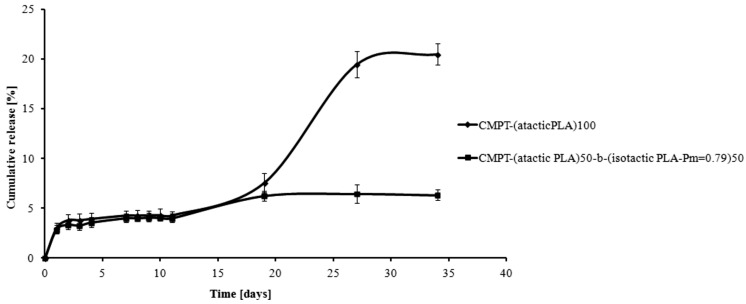
Release of CMPT from the CMPT-PLA conjugates at pH 1.

**Figure 3:**
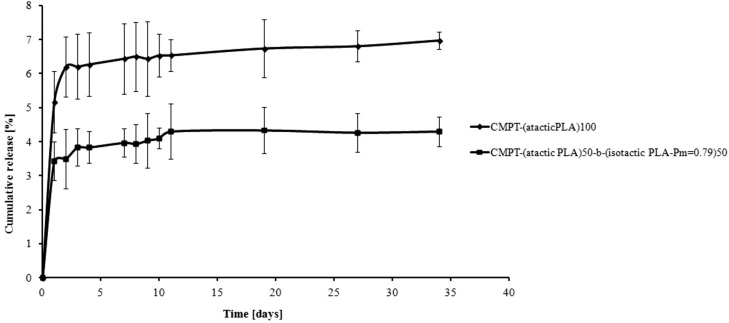
Release of CMPT from the CMPT-PLA conjugates at pH 7.

The *in vitro* kinetic rates for CMPT release from the CMPT-PLA conjugates was determined at pH 7.4 and 1, at 37 °C for 35 days (Figure 2 and Figure 3). The degradation process is rather slow and regular, in comparison with the degradation rates of similar polymeric conjugates. This fact is due to the hydrophobic properties of the PLAs (*M_n_* of the PLAs between 13.5 and 14.0 × 10^3^ Da). **CMPT-PLA-1** (obtained from **PLA-1**) released CMPT faster compared to **CMPT-PLA-2** (obtained from **PLA-2**) (Figure 2 and Figure 3) as 20% of CMPT was released for **CMPT-PLA-1** and 6% for **CMPT-PLA-2** after 35 days of incubation at pH 1 (Figure 2). At pH = 7, 7% and 4% of CMPT was released after 35 days from **CMPT-PLA-1** and **CMPT-PLA-2**, respectively (Figure 3). The lower degradation and hydrolysis rate of **CMPT-PLA-2** is a logical consequence of the increase in crystallinity due to the presence of isotactically enriched PLA blocks. Moreover, as shown in Figure 2 and Figure 3, at pH 1 CMPT is released faster than under neutral conditions, which is attributed to the expected higher hydrolysis rate of the ester groups in this environment. The crucial result presented in Figure 2 concerns the release of CMPT from **CMPT-PLA-1** and **CMPT-PLA-2**, at pH 1. The percentage of CMPT released was about 5% for **CMPT-PLA-1** for first 11 days and reached 20% after following 14 days (between 19–35 days) of incubation process. Noteworthy is the fact that degradation rates of **CMPT-PLA-2**, after initial release of 3.8% of CMPT after 2 days is essentially constant and slow, which shows clearly that microstructure of PLA is crucial for CMPT release kinetics.

## 3. Experimental Section

### 3.1. General Information

Syntheses of PLA was carried out under dry argon using standard Schlenk techniques. Solvents and reagents for PLA syntheses were purified and dried prior to use. Solvents were either dried over potassium (toluene—CHROMASOLV^®^, for HPLC, 99.9% Sigma-Aldrich, Co., Poznan, Poland, hexane—CHROMASOLV^®^, for HPLC, ≥97.0% (GC), Sigma-Aldrich, Co.) or calcium hydride (CH_2_Cl_2_—puriss. p.a., ACS reagent, reag. ISO, ≥99.9% (GC) Sigma-Aldrich, Co.), or purified using a MBRAUN Solvent Purification Systems (MB-SPS-800, MBRAUN, Garching, Germany). *rac*-Lactide was purchased from Aldrich (Poznan, Poland) and further purified by crystallization from anhydrous toluene. 1,8-Diazabicyclo[5.4.0]undec-7-ene (DBU) was purchased from Aldrich, dried over molecular sieves and distilled under argon. (*S,S*)-[Me_2_Ga(μ-OCH(Me)CO_2_Me)]_2_ was synthesized according to the literature [20]. (*S*)-(+)-Camptothecin ((4*S*)-4-ethyl-4-hydroxy-1*H*-pyrano[3',4':6,7]indolizino[1,2-b]quinoline-3,14-(4*H*,12*H*)-dione, ≥90% (HPLC), Sigma Co., Poznan, Poland), 1,4-diazabicyclo[2.2.2]octane (ReagentPlus^®^, ≥99%, Sigma-Aldrich, Co.) and 1,6-diisocyanatohexane (≥98.0% (GC), Fluka Co., Poznan, Poland) were used without further purification. Dichloromethane (anhydrous, ≥99.8%, POCh, Gliwice, Poland), diethyl ether (anhydrous, 99.5%, POCh, Gliwice, Poland) and ethanol (anhydrous, 99.9%, POCh) were used as received. Phosphate-citrate buffer solution (1.0 M, pH 7.00 (25 °C), Na_2_HPO_4_/C_6_H_8_O_7_·H_2_O) and glycine buffer solution ((0.6 M, pH 1.00 (25 °C), NH_2_CH_2_COOH·HCl/NaCl), Chempur Co., Warsaw, Poland) were also used as received. The polymerization products were characterized in the CDCl_3_ or DMSO-d_6_ solution by means of ^1^H-NMR (200, 300 or 400 MHz, Varian, Palo Alto, CA, USA). The FTIR spectra were measured from KBr pellets (model, Perkin-Elmer, Waltham, MA, USA). The amount of released CMPT was quantitatively determined by UV-Vis spectrophotometry (UV‑1202, Shimadzu, Duisburg, Germany) in aqueous buffered solutions at the adsorption maximum of the free drug (λ = at 355 or 368 nm) using a 1 cm quartz cell. The concentration of gallium in the obtained PLAs was determined by atomic absorption spectrometry (Avanta Ultra GPC Z, equipped with an electrothermal atomizer and PAL 4000 autosampler, GBC, Dandenong, Victoria, Australia).

### 3.2. Synthesis of PLA Matrices

(Atactic PLA)_100_ [20] and (atactic PLA)_50_-*b*-(isotactic PLA-*P*_m_=0.79)_50_ [19] were synthesized according to the literature. The structure of (atactic-PLA)_100_ and (atactic PLA)_50_*-b-*(isotactic PLA-*P*_m_=0.79)_50_ was verified by ^1^H-NMR, ^13^C-NMR and homonuclear decoupled ^1^H-NMR spectroscopy (see Supplementary Materials).

(Atactic-PLA)_100_^1^H-NMR (CDCl_3_, 400 MHz); (a) PLA signals: 1.54–1.59 (m, 3H, *CH*CH_3_), 5.12–5.27 (m, 1H, CH*CH_3_*); (b) end groups: 3.73, 3.74 (s, 3H, OCH_3_), 4.36 (br, 1H, CH(*CH_3_)-OH*). *M*_n_ (determined by ^1^H-NMR) 13,400 Da, *P*_m_ = *P*_r_ = 0.5—calculated on the basis of homonuclear decoupled ^1^H-NMR spectra according to Chemberlain *et al.* [22] and on the basis of ^13^C-NMR spectra according to Kasperczyk *et al.* [23] and Abbina *et al.* [24]. (Atactic PLA)_50_*-b-*(isotactic PLA-*P*_m_=0.79)_50_^1^H-NMR (CDCl_3_, 400 MHz); (a) PLA signals: 1.54–1.58 (m, 3H, *CH*CH_3_), 5.12–5.24 (m, 1H, CH*CH_3_*); (b) end groups: 3.73, 3.74 (s, 3H OCH_3_), 4.34 (br. Q, 1H, CH(*CH_3_)-OH*). *M*_n_ (determined by ^1^H-NMR) 13,900 Da, *P*_m_ = 0.65—calculated on the basis of homonuclear decoupled ^1^H-NMR spectra according to Chemberlain *et al.* [22] and on the basis of ^13^C-NMR spectra according to Kasperczyk *et al.* [23] and Abbina *et al.* [24]. For further preparation of CMPT-PLA conjugates PLA was precipitated from a cold diethyl ether and dried under vacuum for 7 days.

### 3.3. Toxicity Assays

#### 3.3.1. Microtox^®^

The Microtox^®^ assay with the luminescent bacteria *Vibrio fischeri* was performed with the lyophilized bacteria purchased from SDI (Newark, DE, USA). The test was performed using disposable glass cuvettes. Samples were incubated at 15 °C for 15 min and the light output of the samples was recorded with a Microtox^®^ M500 analyzer (New Castle, DE, USA). As a diluent and a control 2% NaCl was used.

#### 3.3.2. Protoxkit F^TM^

Protoxkit F^TM^ is a multigeneration protozoan growth inhibition bioassay with the ciliate *Tetrahymena thermophila*. The test is based on the turnover of the substrate (food suspension) into ciliate biomass. While normal proliferating cell cultures clear the substrate suspension in 24 h, inhibited culture growth is reflected by remaining turbidity. The test is based on optical density measurements. The protozoa and the food were obtained from MicroBioTests (Deinze, Belgium). The test was performed in disposable spectrophotometric cuvettes according to the standard operational protocol of the producer. As a diluents and a control deionised water (Milli-Q quality) was used.

#### 3.3.3. Spirotox 

Spirotox test with the protozoan *Spirostomum ambiguum* was performed according to the standard protocol. The test was carried out in disposable, polystyrene multiwell plates (24 wells). Ten organisms were added to each well of the multiwell. The samples were incubated in the darkness at 25 °C for 24 h. Afterwards test responses: different deformations such as shortening, bending of the cell, *etc.*, and lethal response were observed with the use of dissection microscope (magnification of 10). As a diluents and a control Tyrod solution (medium containing microelements: NaCl, KCl, CaCl_2_, MgCl_2_ and NaHCO_3_, NaH_2_PO_4_) was used. In the direct contact test 1 mg of the tested sample was weighted directly to the test containers and poured with 1 mL of appropriate diluent. The organisms were incubated with the suspension of the tested sample. In the extraction test 10 mg of the polymers were incubated with 10 mL of Tyrod solution at 37 °C for 24 h. The organisms were incubated only with extract. One mL of the extract refers to 1.0 mg of the polymer.

### 3.4. Synthesis of Camptothecin Conjugates

CMPT-PLA conjugates were synthesized under an argon atmosphere using two-step process [3]. In the first step, PLA (1 g) was mixed with 1,4-diazabicyclo[2.2.2]octane (0.0025 g) and dissolved in CH_2_Cl_2_ (20 mL). After *ca*. 1 h, 1,6-diisocyanatohexane (0.015 mL) was progressively added into the reactor. Next, the content of the flask was vigorously stirred for 4 h. After this time, the solution of CMPT in CH_2_Cl_2_ was added dropwise into the reactor with the prepolymer under vigorous stirring (at the molar ratio of CMPT to prepolymer equal to 2:1). Next, the reaction mixture was left stirring for an additional 24 h at reflux. Then, it was washed with dilute hydrochloric acid (5% aqueous solution) and distilled water. The product isolated from the organic phase solution were kept under vacuum at room temperature for 2 days.

### 3.5. Camptothecin Release Study from the Macromolecular Conjugates

The *in vitro* release study of CMPT from the synthesized conjugates was investigated by measuring the concentration of CMPT released at pH 1 or 7. Macromolecular conjugates have been dispersed in buffer solutions. All experiments were carried out in triplicate. 20 mg of dried macromolecular conjugate was immersed into 10 mL of buffer solution and incubated at 37 °C, with continuous orbital rotation at 50 [cycles/min]. At predetermined time intervals, a 10 mL samples were withdrawn from the release medium using the filter followed by replace with a 10 mL of a fresh buffer solution. The products were separated by centrifugation (10,000 rpm). The absorption of buffer solution was determined by a UV-Vis spectrophotometer at the absorbance peak with a wavelength at 355 (lactone form) or 368 nm (carboxyl form) [25,26]. The absorbance peak correlated very well with the concentration of CMPT. A linear calibration curve was obtained by measuring the absorption of solutions with predetermined CMPT concentrations. For all the measurements in this study, the absorbance readings were within the calibration range.

## 4. Conclusions

Camptothecin-polylactide conjugates (CMPT-PLA) were synthesized and characterized as macromolecular biodegradable prodrug systems. Polylactides of different microstructures—atactic PLA and (atactic PLA)-*block*-(isotactic PLA)—were used as polymeric matrices in the synthesis of macromolecular conjugates. The rate of release of CMPT from the obtained conjugates depends strongly on the PLA microstructure as well as the pH of the environment. Our results show that modification of PLA microstructures can be a good solution for the modification of the drug-release properties of polymer-drug conjugates, while the rate of release of CMPT from CMPT-PLA conjugates indicates the possibility of using the obtained conjugates for medium- or long-term anti-cancer drug delivery systems. Our present research is focused on a deeper explanation of the influence of PLA microstructure on the drug release properties of PLA based drug conjugates and the synthesis of PLA based conjugates for controlled drug delivery systems.

## Supplementary Materials

Supplementary materials can be accessed at: http://www.mdpi.com/1420-3049/19/12/19460/s1.

## References

[1] Wu S.-F., Hsieh P.-W., Wu C.-C., Lee C.-L., Chen S.-L., Lu C.-Y., Wu T.-S., Chang F.-R., Wu Y.-C. (2008). Camptothecinoids from the seeds of Taiwanese *Nothapodytes Foetida*. Molecules.

[2] Fan H., Huang J., Li Y., Yu J., Chen J. (2010). Fabrication of reduction-degradable micelle based on disulfide-linked graft copolymer-camptothecin conjugate for enhancing solubility and stability of camptothecin. Polymer.

[3] Sobczak M., Oledzka E., Kwietniewska M., Nalecz-Jawecki G., Kołodziejski W. (2014). Promising macromolecular conjugates of camptothecin—The synthesis, characterization and *in vitro* studies. J. Macromol. Sci. Pure Appl. Chem..

[4] Warnecke A., Kratz F. (2003). Maleimide-oligo(ethylene glycol) derivatives of camptothecin as albumin-binding prodrugs: Synthesis and antitumor efficacy. Bioconjugate Chem..

[5] Greenwald R.B., Zhao H., Xia J. (2003). Tripartate poly(ethylene glycol) prodrugs of the open lactone form of camptothecin. Bioorg. Med. Chem..

[6] Bhatt R., de Vries P., Tulinsky J., Bellamy G., Baker B., Singer W., Klein P. (2003). Synthesis and *in vivo* antitumor activity of poly(l-glutamic acid) conjugates of 20(*S*)-camptothecin. J. Med. Chem..

[7] Singer J.W., Bhatt R., Tulinsky J., Buhler K.R., Heasley E., Klein P., de Vries P. (2001). Water-soluble poly-(l-glutamic acid)-Gly-camptothecin conjugates enhance camptothecin stability and efficacy *in vivo*. J. Control. Release.

[8] Cheng J.J., Khin K.T., Jensen G.S., Liu A., Davis M.E. (2003). Synthesis of linear, β-cyclodextrin-based polymers and their camptothecin conjugates. Bioconjugate Chem..

[9] Caiolfa V.R., Zamai M., Fiorino A., Frigerio E., Pellizzoni C., D’Argy R., Ghiglieri A., Castelli M.G., Farao M., Pesenti E. (2000). Polymer-bound camptothecin: Initial biodistribution and antitumour activity studies. J. Control. Release.

[10] Fan N., Duan K., Wang C., Liu S., Luo S., Yu J., Huang J., Li Y., Wang D. (2010). Fabrication of nanomicelle with enhanced solubility and stability of camptothecin based on α,β-poly[(*N*-carboxybutyl)-l-aspartamide]-camptothecin conjugate. Colloids Surf. B.

[11] Zhang W., Huang J., Fan N., Yu J., Liu Y., Liu S., Wang D., Li Y. (2010). Nanomicelle with long-term circulation and enhanced stability of camptothecin based on mPEGylated α,β-poly (l-aspartic acid)-camptothecin conjugate. Colloids Surf. B.

[12] Chun C., Kuh H.-J., Song S.-C. (2012). Injectable poly(organophosphazene)-camptothecin conjugate hydrogels: Synthesis, characterization, and antitumor activities. Eur. J. Pharm. Biopharm..

[13] Ignatius A.A., Claes L.E. (1996). *In vitro* biocompatibility of bioresorbable polymers: Poly(l,dl-lactide) and poly(l-lactide-*co*-glycolide). Biomaterials.

[14] Jelonek K., Kasperczyk J., Li S., Dobrzyński P., Jarząbek B. (2011). Controlled poly(l-lactide-*co*-trimethylene carbonate) delivery system of cyclosporine A and rapamycine—The effect of copolymer chain microstructure on drug release rate. Int. J. Pharm..

[15] Dijkstra P.J., Du H., Feijen J. (2011). Single site catalysts for stereoselective ring-opening polymerization of lactides. Polym. Chem..

[16] Słomkowski S., Penczek S., Duda A. (2014). Polylactides—An overview. Polym. Adv. Technol..

[17] Zhao W., Wang Y., Liu X., Chen X., Cui D., Chen E.Y.-Z. (2012). Protic compound mediated living cross-chain-transfer polymerization of rac-lactide: Synthesis of isotactic (crystalline)-heterotactic (amorphous) stereomultiblock polylactide. Chem. Commun..

[18] Horeglad P., Szczepaniak G., Dranka M., Zachara J. (2012). The first facile stereoselectivity switch in the polymerization of *rac*-lactide-from heteroselective to isoselective dialkylgallium alkoxides with the help of *N*-heterocyclic carbenes. Chem. Commun..

[19] Horeglad P., Litwińska A., Żukowska G.Z., Kubicki D., Szczepaniak G., Dranka M., Zachara J. (2013). The influence of organosuperbases on the structure and activity of dialkylgallium alkoxides in the polymerization of *rac*-lactide: The road to stereo diblock PLA copolymers. Appl. Organomet. Chem..

[20] Horeglad P., Kruk P., Pécaut J. (2010). Heteroselective Polymerization of *rac*-lactide in the presence of dialkylgallium alkoxides: The effect of Lewis base on polymerization stereoselectivity. Organometallics.

[21] Sobczak M., Plichta A., Oledzka E., Jaklewicz A., Kuras M., Cwil A., Kołodziejski W.L., Florjanczyk Z., Szatan K., Udzielak I. (2009). Some spectrometric determination of metals in aliphatic polyester and polycarbonate biomedical polymers. Polimery.

[22] Chemberlain B.M., Cheng M., Moore D.R., Ovitt T.M., Lobkovsky E.B., Coates G.W. (2001). Polymerization of lactide with zinc and magnesium β-diiminate complexes: Mtereocontrol and mechanism. J. Am. Chem. Soc..

[23] Kasperczyk J.E. (1995). Microstructure analysis of poly(lactic acid) obtained by lithium tert-butoxide as initiator. Macromolecules.

[24] Abbina S., Du G. (2014). Zinc-catalyzed highly isoselective ring opening polymerization of *rac*-lactide. ACS Macro Lett..

[25] Amna T., Hassan M.S., Nam K.-T., Bing Y.Y., Barakat N.A.M., Khil M.-S., Kim H.Y. (2012). Preparation, characterization, and cytotoxicity of CPT/Fe_2_O_3_-embedded PLGA ultrafine composite fibers: A synergistic approach to develop promising anticancer material. Int. J. Nanomedicine.

[26] Zhao H., Lee C., Sai P., Choe Y.H., Boro M., Pendri A., Guan S.Y., Greenwald R.B. (2000). 20-*O*-acylcamptothecin derivatives: Evidence for lactone stabilization. J. Org. Chem..

