# Supplementary material for: Polylactide Conjugates of Camptothecin with Different Drug Release Abilities
Source: Molecules. 2014 Nov 25;19(12):19460–70. doi: 10.3390/molecules191219460 (PMC6270997; doi:10.3390/molecules191219460)

Supplementary Materials

Figure S1. ^{1}H-NMR spectrum of (atactic-PLA)$_{100}$.

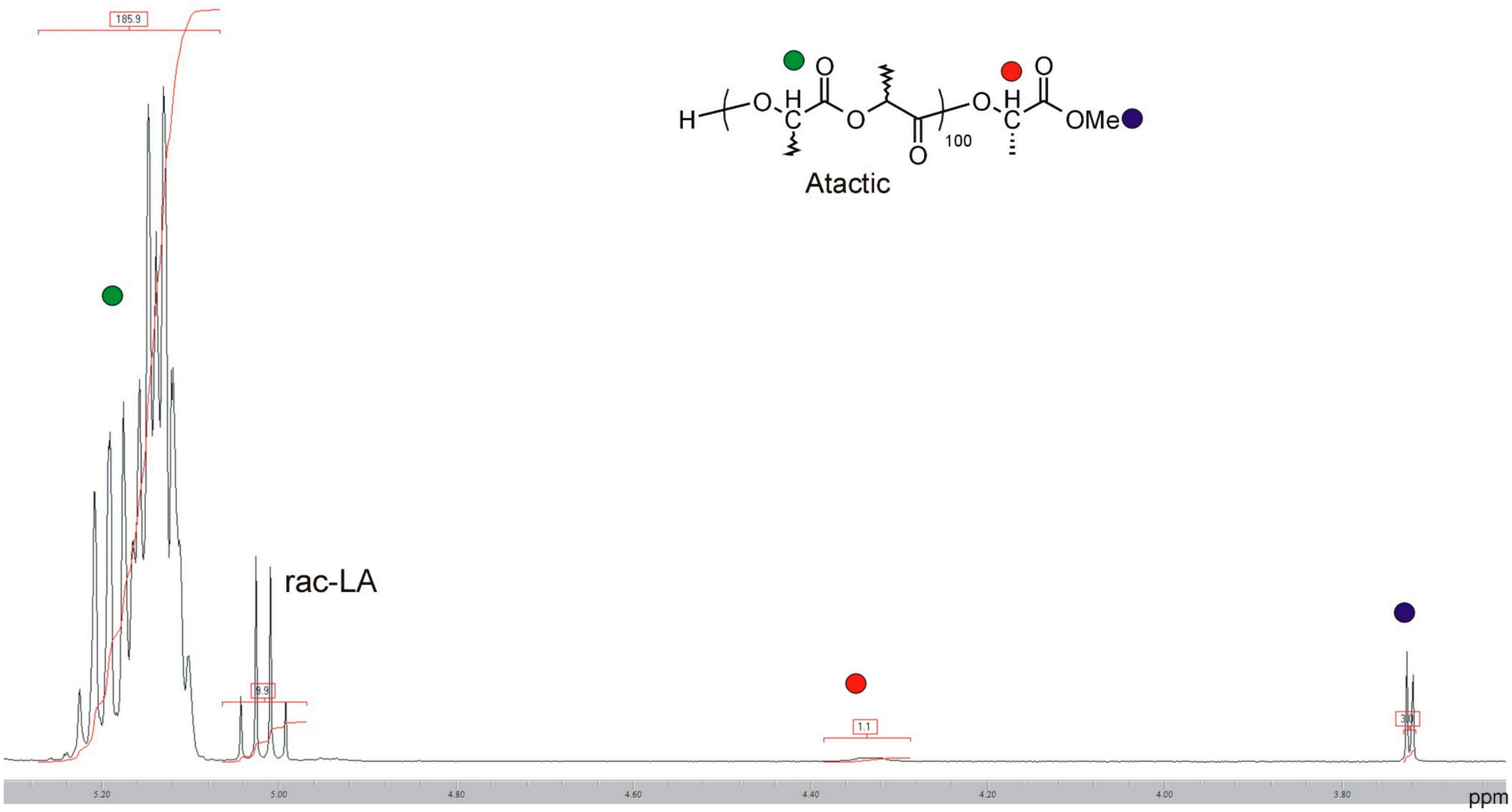

Figure S2. ^{1}H-NMR spectrum of (atactic PLA)$_{50}$-*b*-(isotactic PLA-P_m=0.79)$_{50}$.

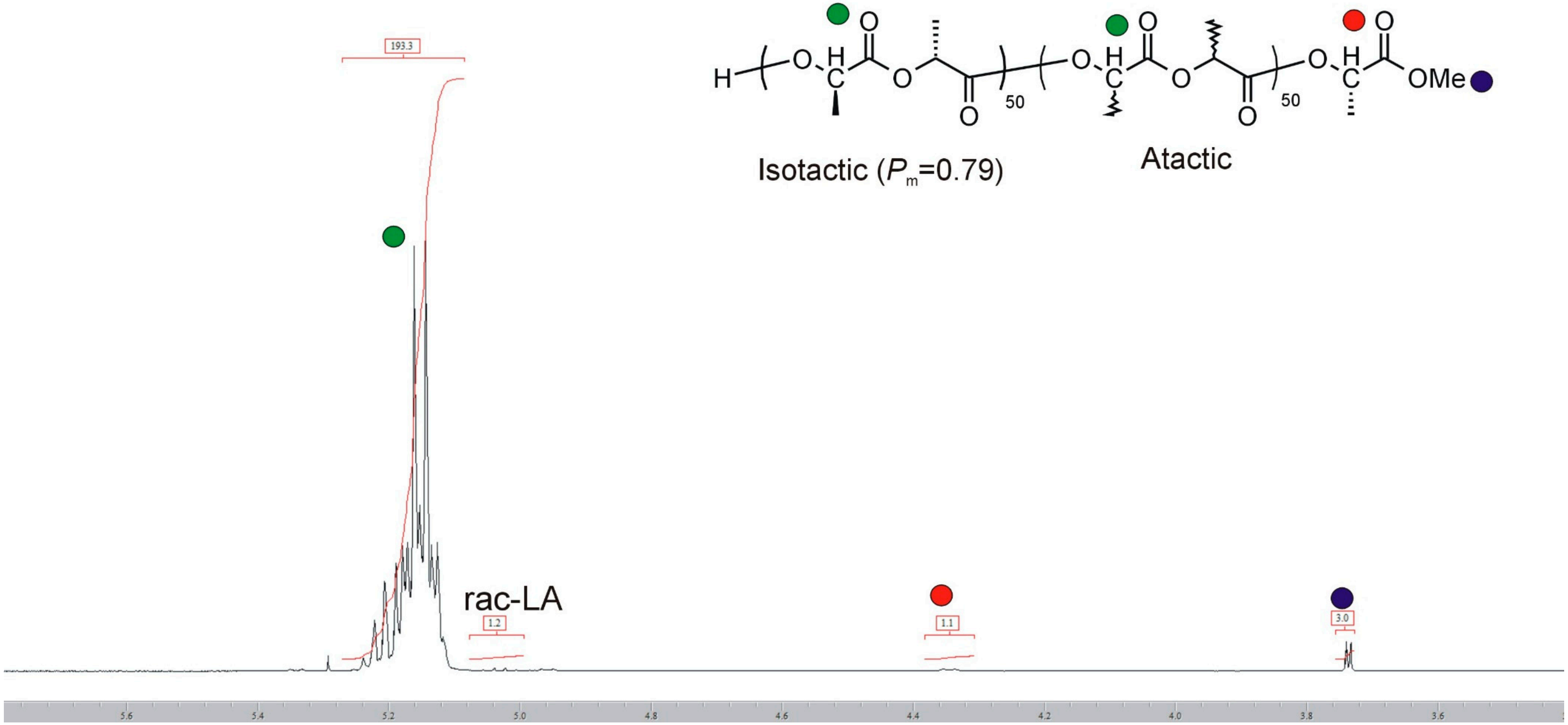

Figure S3. Homonuclear decoupled ^{1}H-NMR spectrum of (atactic-PLA)$_{100}$.

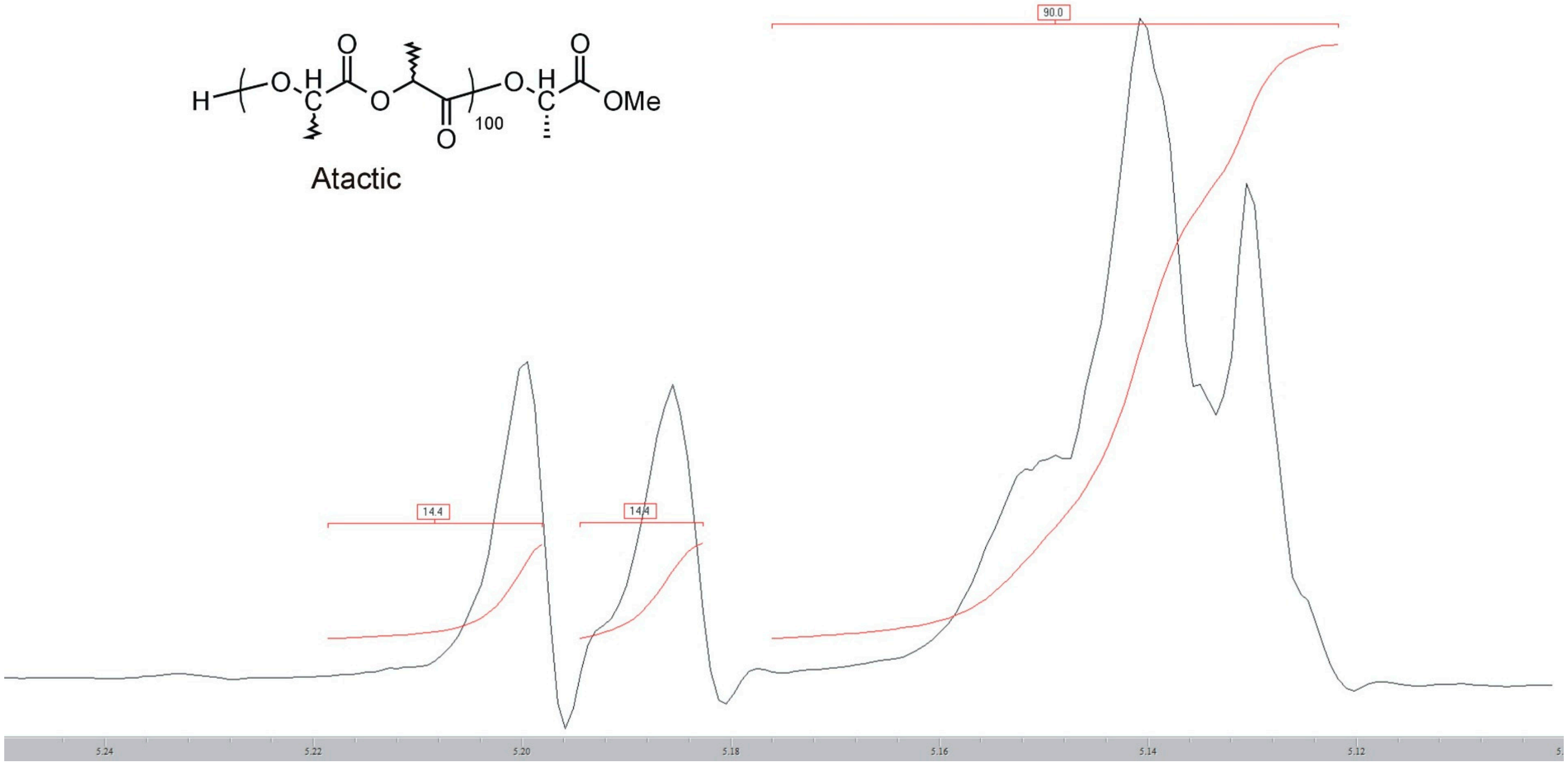

Figure S4. Homonuclear decoupled ^{1}H-NMR spectrum of (atactic PLA)$_{50}$-*b*-(isotactic PLA-P_m=0.79)$_{50}$.

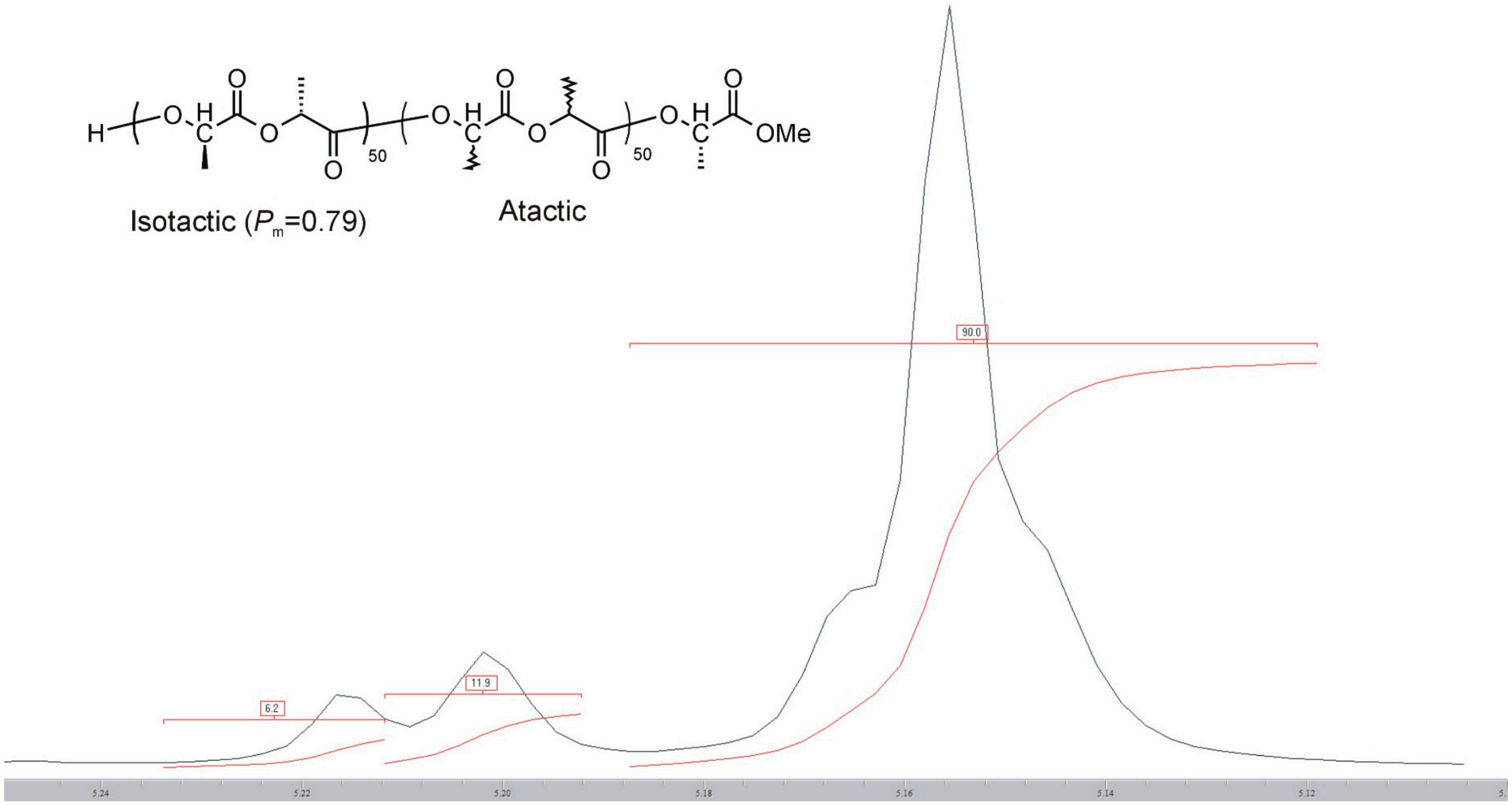

Figure S5. ^{13}C-NMR spectrum of (atactic-PLA)$_{100}$.

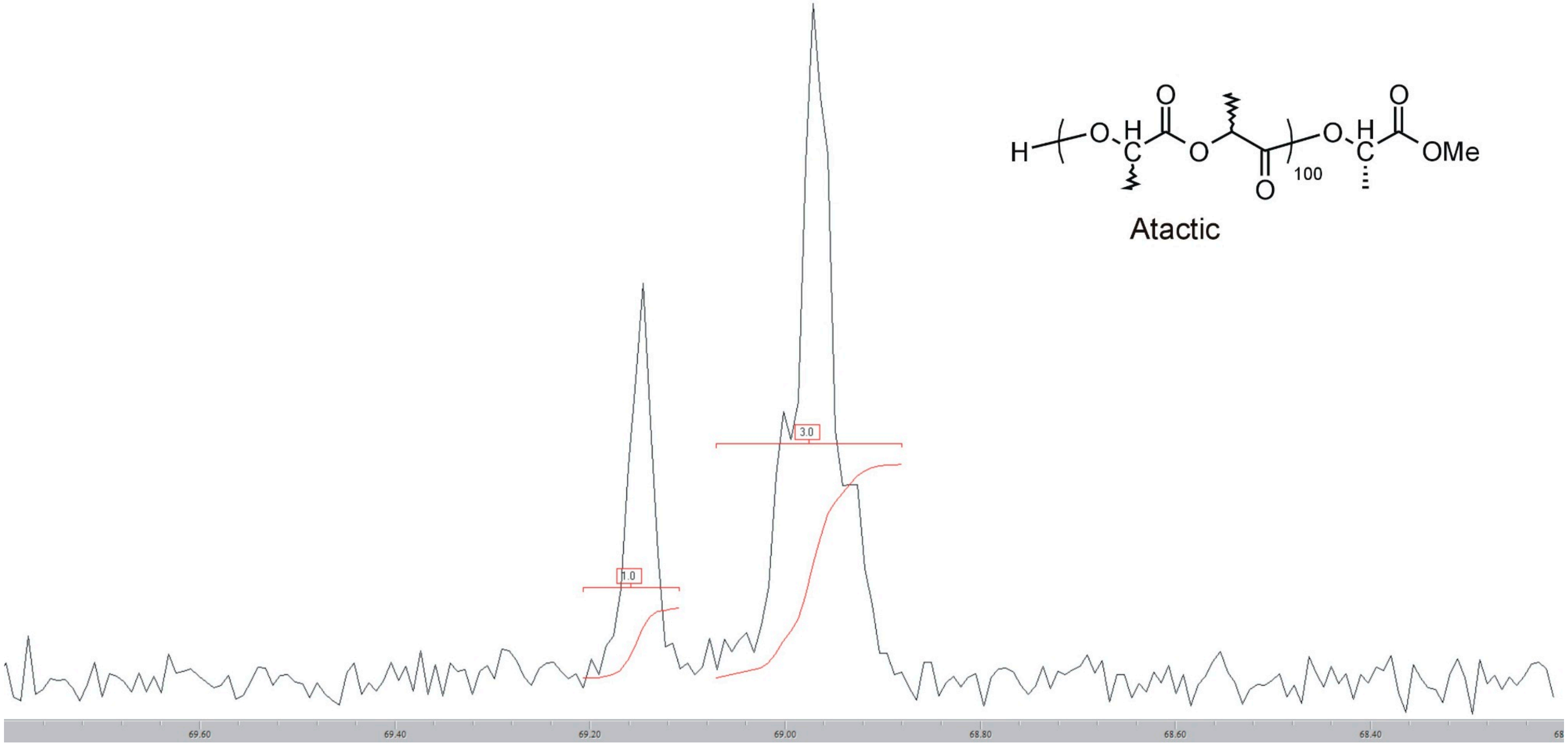

Figure S6. ^{13}C-NMR spectrum of (atactic PLA)$_{50}$-*b*-(isotactic PLA-P_{m}=0.79)$_{50}$.

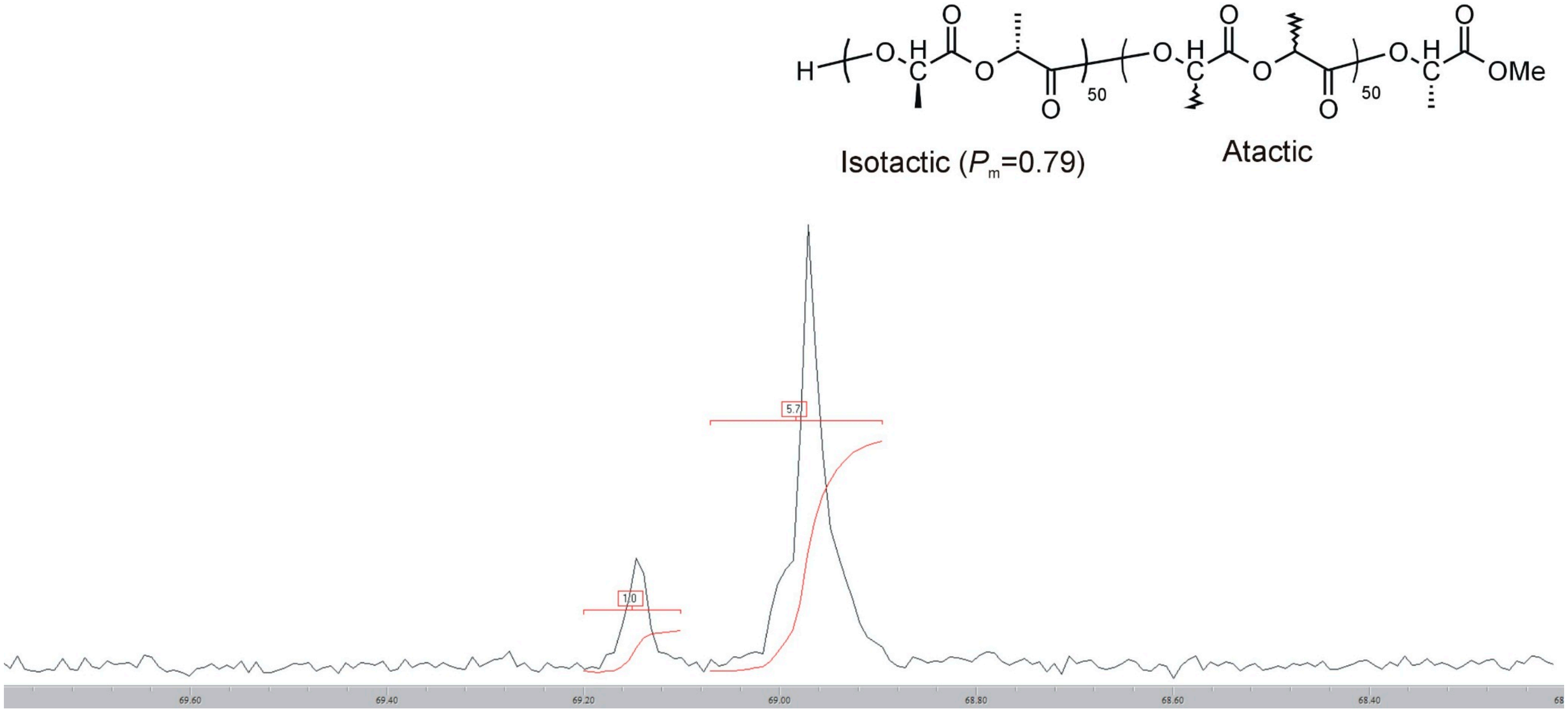

Supplement: Supplementary file 1 [file molecules-19-19460-s001.pdf]
